# Prevalence of Accessory Renal Arteries in Africa: A Systematic Review and Meta‐Analysis Using Anatomical Quality Assurance (AQUA) Checklist

**DOI:** 10.1002/hsr2.71751

**Published:** 2026-01-14

**Authors:** Seid Mohammed Abdu, Hussen Abdu, Endris Seid, Ebrahim Msaye Assefa

**Affiliations:** ^1^ Department of Biomedical Science, College of Medicine and Health Science Wollo University Dessie Ethiopia

**Keywords:** accessory renal artery, Africa, pattern, prevalence, renal artery variation, systematic review and meta‐analysis

## Abstract

**Background and Aims:**

Variations of accessory renal arteries are different in different population groups, but comprehensive reviews for the ethnically diverse continent of Africa are lacking. Therefore, this review aimed to describe the prevalence of accessory renal arteries in the African population based on specimens from cadaveric, CT angiography, and kidney transplant intraoperative reviews.

**Methods:**

By searching PubMed, Google Scholar, Hinari, and websites (Google, Yahoo), the renal arterial vasculature of 2451 study subjects and 4882 kidneys were retrieved from 17 studies. Anatomical variations of accessory renal arteries were categorized based on prevalence, sex, symmetry, laterality, and termination in the superior pole, hilum, and inferior pole of the kidneys.

**Results:**

Accessory renal arteries were found in 19.7% of study subjects, with 70% of these being males. Additionally, 13.9% of kidneys had accessory renal arteries, with 77.9% of these variations being unilateral. The most common pattern discovered was the presence of single accessory arteries (80.2%). The inferior pole was the predominant point of entry (42%), followed by hilar arteries (39%). Furthermore, a significant difference was observed between the prevalence of left‐ and right‐sided accessory arteries, with a dominance of left‐sided arteries (57.1%).

**Conclusion:**

Accessory renal arteries exhibit high prevalence, particularly among males, with the inferior pole as the primary entry point and left‐sided dominance. However, the significant heterogeneity and the lack of data from most countries necessitate cautious interpretation. In light of these variations, these findings point out the importance of these reviewed articles as essential references for renal transplantation and other urological procedures.

## 1 Introduction

The kidneys usually get their blood supply from a single renal artery arising from the abdominal aorta between lumbar vertebrae I and II and terminating in the kidney through the hilum [1]. However, variations in kidney blood supply are common due to the kidneys' migration cranially from the pelvis. As they ascend, they typically receive new branches from a higher part of the aorta, causing the caudal vessels to regress and disappear. When regression fails, accessory renal arteries (ARAs) can develop [2].

The incidence of ARAs is of clinical and scientific interest, not only because of their clinical relevance when performing renal surgery or transplantation [3]. In addition, knowledge of their existence is also important for therapies in patients with resistant hypertension [3] and lower polar arteries originating from the aorta, which can cause hydronephrosis [4].

The presence of ARAs can be explained by different terminology, like additional renal artery [5, 6, 7], aberrant [8], supernumerary [9, 10], supporting [11, 12], and multiple [13, 14]. We prefer to use the term accessory renal arteries (ARAs) because it is widely used in the literature [5, 15, 16, 17, 18, 19]. The term “accessory” however should not be interpreted as non‐essential. These arteries are end arteries, meaning that any disease, blockage, or ligation affecting them leads to a reduced blood supply to the associated segment of kidney tissue, resulting in necrosis and infarction.

Variations in the number, source, and course of the renal arteries are common, the most common variation being the ARAs [20]. It appears in about 25%–30% of the general population and represents the persistence of the embryonic pattern [21]. These vascular variations have been broadly reviewed by Matta et al. [22], who examined various renal vascular structures, including arteries and veins; however, their study did not specifically focus on the detailed anatomical characteristics of ARAs. Similarly, Triantafyllou et al. [23] conducted a review that included only seven African studies, limiting its ability to accurately represent the prevalence and patterns of ARAs across the continent. Furthermore, existing primary studies report inconsistent prevalence rates, likely due to ethnic and regional differences [24]. This highlights a critical gap in the literature and emphasizes the need for a comprehensive systematic review to synthesize available data and enhance understanding of ARAs in Africa. Therefore, this review aimed to determine the prevalence of ARAs in Africa through a systematic review and meta‐analysis.

## 2 Methods

### 2.1 Protocol Registration

To provide comprehensive information, we conducted a systematic review and meta‐analysis on the prevalence of ARAs in Africa. This review was conducted following a registered protocol in the International Prospective Register of Systematic Reviews (PROSPERO) under the registration number [CRD42024548407].

### 2.2 Search Strategy

This systematic review and meta‐analysis were reported by following the Preferred Reporting Items (PRISMA) guidelines (PRISMA checklist) [25]. A comprehensive literature search was conducted across multiple electronic databases, including PubMed, Google Scholar, Hinari, as well as general search engines (Google and Yahoo), to identify additional gray literature, covering studies published up to April 30, 2024. The search strategy was executed in three stages. In the first stage, relevant Medical Subject Headings (MeSH) and relevant keywords were identified based on the review of the literature. Then full searches were conducted in selected database using a combination of MeSH terms and keywords, kinked by Boolean operators ((OR, AND). Finally, the bibliography of pertinent studies was reviewed, and additional eligible studies were sought from university websites. The search strategies were then conducted in the selected databases using a combination of MeSH terms and keywords liked by Boolean operators. The search strategies included terms such as “prevalence, incidence, magnitude, epidemiology, and proportion”, combined with phrase like “accessory renal arteries”, “supernumerary renal arteries”, “supporting renal arteries”, “multiple renal arteries”, “additional renal arteries”, “aberrant renal arteries”, and “anatomical variation of renal arteries” in the title or abstract. The search was filtered by country name, English Language, free full‐text availability and publication dates from 2000 to April 2024 (Supporting Information S1: Appendix 1).

## 3 Inclusion and Exclusion Criteria

This review includes observational studies which report the prevalence of ARAs in a population of any age group, specifically in Africans. Only studies conducted and published in the English Language over the past 24 years were considered. The studies must be in line with the diagnostic methods, such as cadaver dissection, imaging, or intraoperative procedures, to identify these variations. The primary outcome of interest was the prevalence of ARAs. Studies excluded from this review were case reports, case series, and any research that did not provide sufficient data to estimate the prevalence of ARAs.

### 3.1 Selection Process

The study selection for this systematic review and meta‐analysis was carried out in two distinct phases. Initially, a comprehensive search of relevant electronic databases was performed to identify potential articles, followed by the removal of duplicates using Rayyan. The remaining records were then screened based on titles and abstracts to determine their relevance according to predefined inclusion and exclusion criteria. Studies deemed potentially relevant underwent a full‐text review for eligibility assessment. Two reviewers (S.M.A. and E.M.A.) independently evaluated each study's eligibility. Any disagreements were discussed, and the inclusion/exclusion criteria were revisited to guide the resolution. When consensus could not be achieved, a third reviewer was consulted to make the final decision.

### 3.2 Data Extraction and Quality Assessment

Data extraction was independently performed by the same two reviewers using a standardized Microsoft Excel spreadsheet. Key variables extracted included the first author's name, year of publication, and country of study, diagnostic modality used, prevalence and pattern of ARAs, sample size, and the number of ARAs reported. Any discrepancies between reviewers were resolved through discussion. To evaluate methodological rigor, the Anatomical Quality Assurance (AQUA) checklist [26], which is applicable to anatomical studies (Supporting Information S2: Appendix).

### 3.3 Outcome of Interest

The main outcome of interest was the prevalence of ARAs among the total number of assessed study subjects. The prevalence was calculated by dividing the number of ARAs by the total number of subjects and kidneys.

### 3.4 Statistical Analysis

Multilevel pooled analysis of anatomical variation of ARAs, accompanied by a corresponding 95% CI, was calculated using the random‐effects, inverse variance method. The heterogeneity of studies was checked using Cochran's Q test and *I* ^2^ test statistics. To explore potential sources of heterogeneity, a pre‐defined subgroup analysis was performed based on the means of diagnosis, and countries where the studies were conducted. Statistical analyses were performed using STATA version 17 (StataCorp LLC, College Station, TX, USA). In addition, publication bias was assessed using MetaXL software through Doi plots and the Luis Furuya‐Kanamori (LFK) index, which are recommended tools for meta‐analyses of proportion data.

## 4 Result

**Figure 1:**
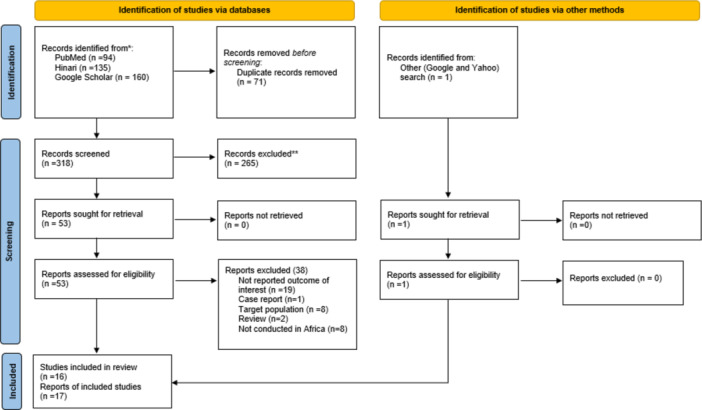
Depicts the schematic flow of study selection steps for anatomical variations of the ARA in Africa.

**Figure 2:**
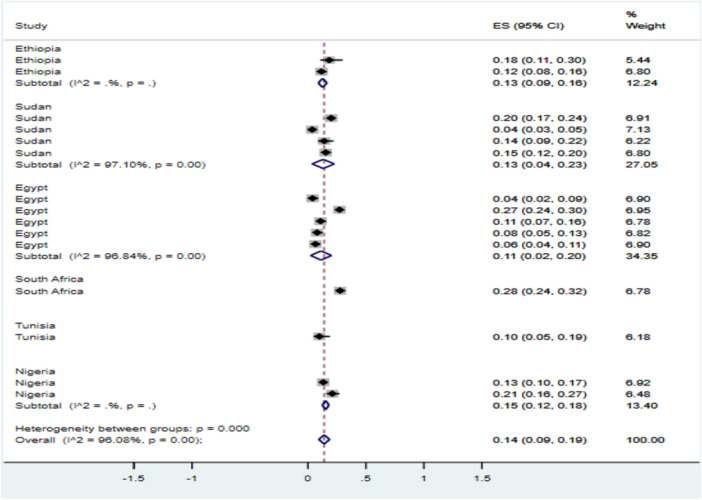
Forest plot showing the subgroup analysis by countries for the pooled prevalence of ARA in Africa, 2024.

In our initial search, 389 publications were identified from databases. After removing 71 duplicates, 318 unique articles remained for screening. Of these, 265 were excluded based on their titles and abstracts for not meeting the eligibility criteria. The remaining 53 full‐text articles were assessed, of which 15 met the inclusion criteria, while the other 38 were excluded. The reasons for excluding these 38 articles are presented in Figure 2. Among the 15 included articles, one contained two distinct reports, resulting in a total of 16 reports. Additionally, one more report was identified through manual searches on Google and Yahoo, bringing the final total to 17 reports included in the review (Figure 1).

### 4.1 Characteristic of Studies

This meta‐analysis included 17 studies with a combined total of 2451 study participants, examining 4882 kidneys for anatomical variations of ARAs. The studies were conducted across seven African countries: three in Ethiopia, four in Sudan, five in Egypt, two in Nigeria, and one each in Tunisia, Libya, and South Africa. All the studies utilized an observational descriptive design. The main diagnostic methods employed were cadaveric examinations, CT angiography, and kidney transplant records (Table 1).

**Table 1:** Description of studies included in the meta‐analysis for the prevalence of ARA among study subjects in Africa.

First author name and year	Countries	Sample size	Modality of diagnosis	N° of subjects with ARA	Prevalence of ARA in study subjects (95% CI)	N° of male and female with ARA				Prevalence of ARA between sexes			
						Male		Female		Male		Female	
Abba et al. 2015 [27]	Ethiopia	16	cadaver	3	18.8% (6.6, 43)	3		—		100%		—	
Zelalem et al. 2017 [28]	Ethiopia	30	cadaver	10	33.3% (19.2, 51.2)	—		—		—		—	
Gebre et al. 2020 [29]	Ethiopia	120	Image	28	23.3% (16.7, 31.7)	19		9		67.9%		32.1%	
Amal et al. 2011 [1]	Sudan	301	Both and image cadaver	110	36.5% (31.3, 42.1)	65		45		59.1%		40.9%	
Safaa et al. [30]	Sudan	400	Image	24	6% (4.1, 8.8)	15		9		62.5%		37.5%	
Mugahid et al. 2016 [31]	Sudan	50	Cadaver	11	22% (12.8, 35.2)	11		—		100%		—	
Mugahid et al. 2016 [31]	Sudan	150	Image	40	26.7% (20.2, 34.3)	40		11		75%		25%	
Hassan et al. 2015 [32]	Egypt	63	Cadaver	3	4.8% (1.6, 13.1)	1		2		33.3%		66.7%	
Hoda et al. 2004 [33]	Egypt	390	Both and image cadaver	106	27.2% (23, 31.8)	93		13		87.7%		12.3%	
Asmaa et al. 2019 [34]	Egypt	106	Image	20	18.9% (12.6, 27.4)	7		13		35%		65%	
W. Sameh et al. 2010 [35]	Egypt	89	Image	7	7.9% (3.9, 15.4)	—		—		—		—	
Ahmed S. et al. 2017 [36]	Egypt	100	Image	12	12% (7, 19.8)	—		—		—		—	
Shrifa M. et al. 2022 [37]	Libya	100	Image	6	6% (2.8, 12.5)	—		—		—		—	
O.C. Famurewa et al. 2016 [38]	Nigeria	200	Image	56	28% (22.2, 34.6)	37		19		66.1%		33.9%	
Abayomi A. et al. 2021 [39]	Nigeria	100	Image	32	32% (23.7, 41.7)	—		—		—		—	
Pooled prevalence	—	—		—	19.8% (13.6, 26.1)	—		—		70.1% (58.1, 77.1)		36.4% (23.9, 49)	

### 4.2 Risk of Bias Assessment

The methodological quality of the included studies was assessed using the Anatomical Quality Assurance (AQUA) checklist, which evaluates five domains with a total maximum score of 25. Out of the 17 assessed studies, seven studies (41.2%) were classified as having a low risk of bias, nine studies (52.9%) had a moderate risk of bias, and one study (5.9%) was found to have a high risk of bias. These findings indicate that while the majority of studies demonstrated acceptable methodological rigor, a considerable number still exhibited moderate risk, suggesting variability in study quality across the included literature.

### 4.3 The Prevalence of the ARAs Among Study Subjects

Out of the 17 reviewed articles, 15 studies reported the number of subjects with ARA. These 15 studies included a total of 2215 subjects, with 468 identified as having ARA. This resulted in a pooled prevalence of 19.8% (95% CI: 13.6–26.1) in study subjects. Among patients with ARA, 70.1% were males compared to 36.4% females. The inverse variance *I*² was 94% with *p* < 0.001, indicating significant heterogeneity among the studies. Moreover, the prevalence of ARA among study subjects varied significantly by region, with the lowest prevalence reported in Egypt at 4.8% and the highest in Sudan at 36.5%. (Table 1).

### 4.4 The Prevalence of ARA Among Kidneys

A total of 4882 kidneys were identified across 17 articles. However, only 15 of these articles, involving a total of 4650 kidneys, examined the presence of ARAs, with 729 kidneys identified as having ARAs. According to this data, the pooled prevalence of kidneys with ARAs among the observed kidneys was 14% (95% CI: 9.3–18.7). Furthermore, 12 studies out of the 15 reviewed articles reported on the symmetry of ARAs. Based on these, the prevalence of unilateral symmetry was 77.6% (95% CI: 69.4–86.4), while the prevalence of bilateral ARA was determined to be 25.7% (95% CI: 17.1–34.3). Among the kidneys with ARAs reported in 15 articles, 14 of these articles provided data on the number of ARAs (single vs. multiple). Based on this, 91% (95% CI: 86.1, 95.9) of kidneys had a single ARAs, while 12.9% (95% CI: 7.5, 18.3) showed double ARAs, and 2.5% had triple ARAs (Table 2).

**Table 2:** Description of studies for the prevalence and pattern of ARAs per studied kidneys.

First author name and year	Sample size	N° of kidneys	N° of kidney with ARA	Prevalence of kidney with ARA out of total in kidneys	Single	Double	Symmetry	
							Unilateral	Bilateral
Zelalem A. et al. 2017 [28]	30	60	11	18.3% (10.6–29.9)	72.7%	27.3%	80%	20%
Gebremickael A. et al. 2020 [29]	120	240	28	11.7% (8.2–16.3)	100%	—	100%	—
Amal Y. et al. 2011 [1]	301	549	110	20% (16.9–23.6)	70.9%	29.1%	—	—
Safaa M. et al. [30]	400	794	29	3.7% (2.6–5.2)	100%	—	65.5%	34.5%
Mugahid A. et al. 2016 [31]	50	100	14	14% (8.5–22.1)	92.9%	7.1%	57.1%	42.9%
Mugahid A. et al 2016 [31]	150	300	46	15.3% (11.7–19.8)	93.5%	6.5%	73.9%	26.1%
S.S. Hassan et al. 2015 [32]	63	126	5	4% (1.7–9)	80%	20%	100%	—
Hoda M. et al. 2004 [33]	334	780	212	27.2% (24.2–30.4)	93.4%	6.6%	73.6%	26.4%
Asmaa A. et al. 2019 [34]	106	212	23	10.8% (7.3–15.8)	100%	—	82.6%	17.4%
W. Sameh et al. 2010 [35]	89	89	14	7.9% (4.7–12.8)	92.9%	7.1%	—	—
Ahmed S. et al. 2017 [36]	100	200	13	6.5% (3.8–10.8)	100%	—	84.6%	15.4%
K.S. Satyapal et al. 2000 [6]	236	440	122	27.7% (23.8–32.1)	83.6%	16.4%	82.2%	8.2%
Abderrazak B. et al. 2021 [40]	71	142	7	9.9% (4.9–19)	57.1%	42.9	—	—
O.C. Famurewa 2016 [38]	200	400	53	13.2% (10.3–16.9)	—	—	73.6%	26.4%
Abayomi A. et al. 2021 [39]	100	200	42	21% (15.9–27.2)	92.5%	7.5%	52.4%	47.6%
Pooled prevalence	—	—	—	14% (9.3–18.7)	91%	12.9%	77.6%	25.7%

Out of the 17 articles reviewed, 14 studies provided data on the laterality of ARAs, while 13 studies reported on the termination points of ARAs. In total, 843 ARAs were included in the laterality analysis, and 689 ARAs were counted in the analysis of termination points. Regarding laterality, left‐sided ARAs constituted 57.1% (95% CI: 52.3, 61.9), whereas right‐sided ARAs comprised 43.7% (95% CI: 40.4, 47). Concerning their termination points within the kidneys, 39% (95% CI: 24, 53.9) of ARAs were observed entering through the hilum and are termed hilar ARAs, 42.4% (95% CI: 29.2, 5.5) entered via the inferior pole of the kidney, referred to as inferior polar ARAs, while 20.5% (95% CI: 13.9, 27.1) entered through the superior pole of the kidney, known as superior polar ARAs (Table 3).

**Table 3:** Prevalence of the laterality and termination point of ARA.

Author name	N° of ARA	Laterality of ARA		Termination point of ARA		
		Right	Left	Superior polar	Hilum	Inferior polar
Abba S. et al. 2015 [27]	3	—	100%	—	—	100%
Zelalem A. et al. 2017 [28]	13	23.1%	76.9%	—	15.4%	61.5%
Gebremickael A. et al. 2020 [29]	28	—	—	39.3%	10.7%	50%
Amal Y. et al. 2011 [1]	207	41.1%	58.9%	32.9%	53.6%	13.5%
Safaa M. et al. [30]	29	58.6%	41.4%	—	—	—
Mugahid A. et al. 2016 [31]	15	60%	40%	6.7%	66.7%	26.7%
Mugahid A. et al. 2016 [31]	47	44.7%	55.3%	14.9%	17%	68.1%
S.S. Hassan et al. 2015 [32]	6	33.3%	66.7%	16.7	50%	33.3%
Hoda M. et al. 2004 [33]	229	44.5%	55.5%	15.7%	74.2%	10%
Asmaa A. et al. 2019 [34]	23	52.3%	47.8%	8.7%	47.8%	43.5%
W. Sameh et al. 2010 [35]	15	33.3%	66.7%	—	26.7%	—
Ahmed S. et al. 2017 [36]	13	—	—	30.8%	46.2%	23.1%
K.S. Satyapal et al. 2000 [6]	142	42.3%	57.7%	—	—	—
Abderrazak B. et al. 2021 [40]	10	40%	60%	—	—	—
O.C. Famurewa 2016 [38]	60	45%	55%	25%	23.3%	51.7%
Abayomi A. et al. 2021 [39]	44	47.7%	52.3%	18.2%	38.6%	43.2%
Pooled prevalence		43.7% (40.4, 47)	57.1% (52.3, 61.9)	20.5%	39%	42.4%

### 4.5 Subgroup Analysis of ARA

Subgroup analysis of kidneys with ARAs, as a proportion of the total kidneys, was carried out by the study country. The results showed that the pooled prevalence of kidneys with ARAs in Ethiopia was 13% (95% CI: 9, 16), in Sudan it was 13% (95% CI: 4, 23), and in Egypt 11% (95% CI: 2, 20). A high level of heterogeneity was present in studies carried out in Sudan and Egypt (Figure 2).

**Figure 3:**
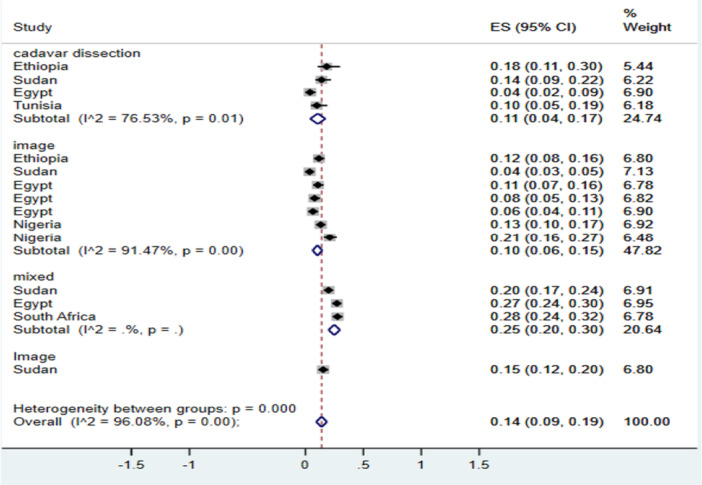
Forest plot showing the subgroup analysis by means of diagnosis for ARA.

Furthermore, subgroup analysis of kidneys with ARAs was conducted based on the means of diagnosis. The pooled point estimate prevalence of kidneys with ARA in cadaver dissection was 11% (95% CI: 4, 17), while in imaging studies, it was 10% (95% CI: 6, 15). Additionally, in studies utilizing both imaging and cadaver dissection, the prevalence of ARA was higher at 25% (95% CI: 20, 30) (Figure 3).

### 4.6 Assessment of Publication Bias

**Figure 4:**
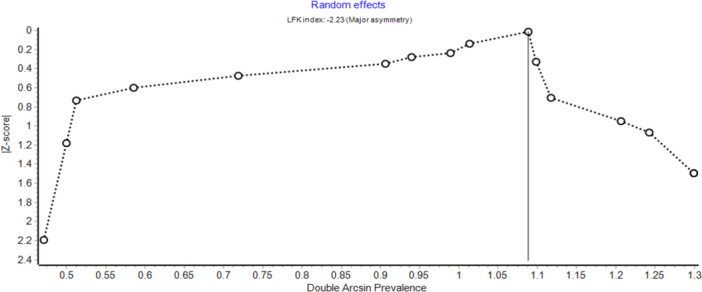
Doi plot assessing publication bias in the pooled prevalence of ARAs among study subjects.

**Figure 5:**
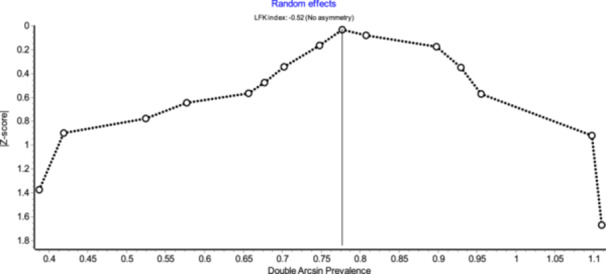
Doi plot assessing publication bias in the pooled prevalence of ARAs among reviewed kidneys.

Publication bias was assessed using the Doi plot and the Luis Furuya‐Kanamori (LFK) index in MetaXL. The analysis showed a high level of asymmetry for studies reporting ARAs based on subjects, with an LFK index of −2.23, indicating substantial publication bias (Figure 4). In contrast, studies that reported ARAs based on kidneys showed an LFK index of −0.53, which falls within the range of no asymmetry, suggesting no evidence of publication bias in that group (Figure 5).

## 5 Discussion

The prevalence and pattern of ARA vary greatly among populations. This analysis, therefore, aimed to pool the results of anatomical variations of ARAs, which are important for clinicians due to their significant roles in renal transplantation and other urological procedures. Occasionally, ARAs might cause hydronephrosis, necessitating clinical approaches to manage it.

The prevalence of ARA shows a wide inconsistency, ranging from 4% in Malaysia [41] to 59.5% in India [42]. Our analysis found ARA in 19.8%, which is consistent with the findings of a previous global systematic review that reported an overall prevalence of 19% [43] and a population‐based study in Australia (22%) [44]. Therefore, given this high prevalence of ARA in our analysis (19.8%), it is crucial to carefully assess renal anatomy prior to procedures involving urological procedures or renal transplantation. Surgeons should be prepared for the potential presence of these ARAs and adapt their approach accordingly.

In our meta‐analysis, we found that among samples with ARAs, 70.1% were males, compared to 36.4% females. This finding aligns with studies conducted in Europe involving 1357 individuals reported a significantly higher prevalence of ARAs in males than females [45]. Similarly, a study from southern India found ARA prevalence in 30% of males versus 11% in females [46]. A study from Iran also reported more males than females (52.7% vs. 47.3%), though the difference was less pronounced [47]. However, not all studies demonstrated a clear male predominance. In a study conducted among 100 healthy individuals from the North Indian population (16 males and 84 females), the prevalence of ARAs was equal between sexes, 25.0% in both males and females [48]. This lack of difference may be attributed to the marked female predominance in the sample, which could have influenced the observed distribution and masked potential sex‐based variations.

Our meta‐analysis revealed a pooled prevalence of ARAs per kidney of 14.0% (95% CI: 9.3%–18.7%), which is lower than the 21.1% (95% CI: 19.25%–23.01%) reported in the recent global systematic review by Triantafyllou et al. (2024) [23]. This discrepancy may be attributed to differences in population demographics, genetic backgrounds, or methodological approaches across studies. Notably, our analysis focused exclusively on studies from Africa, which may reflect regional anatomical variation compared to the more diverse global data set analyzed by Triantafyllou and colleagues. In addition, variations in imaging modalities, cadaveric dissection techniques, and sample sizes across studies may have influenced the reported prevalence. Despite these differences, both studies underscore the relatively high occurrence of ARAs and highlight the importance of recognizing these vascular variants in clinical practice.

In this review, a total of 843 ARAs were analyzed for the laterality. Notably, 57.1% of these ARAs were found on the left side, indicating a left‐sided predominance. This finding is consistent with a study conducted in Iran, which reported a slightly higher prevalence of ARAs on the left side (50.59%) [47]. Similarly, a study of the Caribbean population observed a higher occurrence of ARAs in the left kidneys (56.33%) than in the right [49]. In contrast, a study from India reported a higher incidence of ARAs on the right side compared to the left [50]. These discrepancies highlight the potential influence of ethnic, genetic, or regional anatomical variability on the laterality of ARAs. The findings of this review are therefore important for nephrologists, as the left kidney is often preferred over the right for transplantation due to its longer vein, easier access, and better surgical field. However, the predominance of ARAs on the left suggests that additional criteria should be considered in surgical decision‐making for procedures such as donor nephrectomies and transplantations.

Out of the 689 ARAs identified, 42.4% were revealed to have an inferior polar artery entering the kidney through its inferior pole. It appears to be of greater clinical significance than hilar and superior polar arteries because the upper section of the ureter relies significantly on blood supply from a branch of the inferior polar artery, if present [49] and there's a documented risk of uretero‐pelvic obstruction when the inferior polar artery originates from the aorta and traverses the ureter either anteriorly or posteriorly, leading to complications such as hydronephrosis and pyelonephritis [11].

The clinical significance of ARAs adds valuable context to our anatomical findings. ARAs are not merely anatomical curiosities; they have important clinical implications, especially in renal transplantation and urological surgery. Studies have shown that the presence of ARAs in kidney transplant recipients is associated with higher complication rates, including increased risk of vascular events such as thrombosis and bleeding, particularly in patients with underlying atherosclerosis [51]. Additionally, ARAs have been linked to delayed graft function due to technical challenges during arterial anastomosis [23]. Moreover, ARAs may compromise blood supply to the ureter, potentially leading to ureteral ischemia or necrosis, which can result in poor graft outcomes [51]. When an ARA supplies the uretero‐pelvic junction, surgical injury or ligation may cause hydronephrosis or urinary obstruction [11]. From a broader clinical perspective, the presence of ARAs has also been associated with resistant hypertension [52], further emphasizing their importance in both nephrology and vascular medicine.

In our review, we observed significant heterogeneity among the studies. We conducted subgroup analyses based on countries and methods of diagnosis to identify potential sources of this heterogeneity. However, the subgroup analysis did not explain the observed variability. Furthermore, the lack of data from many African countries raises concern about the generalizability of the results. The terminology referring to ARAs other than the main artery is highly confusing. Therefore, there is a critical need for standardization in nomenclature to address this confusion. Standardization will not only help in accurately reporting the prevalence of ARA but also in alleviating the difficulties associated with varying terminology across studies. By establishing consistent terminology, researchers and clinicians can better communicate findings and facilitate a clearer understanding of renal artery anatomy, ultimately improving patient care and outcomes. We acknowledge this as a limitation of our study, and readers should interpret the pooled estimates with this consideration in mind.

### 5.1 Limitation of the Study

Our inclusion criteria were limited solely to English‐language studies, as it could result in the omission of valuable study data in languages other than English. This limitation has the potential to impact the comprehensiveness and generalizability of our findings.

## 6 Conclusion

The incidence of ARAs was identified in a significant proportion of individuals across the included studies, with a male predominance. At the kidney level, the pooled prevalence of ARAs was 14%, with most arteries occurring unilaterally. A significant difference was observed between the left and right sides, with a predominance of left‐sided ARAs. Regarding the point of termination, the inferior pole was the most common site of entry, followed by the renal hilum. Knowledge of the presence and dominance of ARAs is crucial, as they can influence the management of renal transplantation and urological procedures. Finally, further research is needed to enhance our understanding of these variations and their clinical implications.

## Author Contributions

**Seid Mohammed Abdu:** conceptualization, investigation, writing – original draft, methodology, writing – review and editing, validation, software, formal analysis, data curation, supervision. **Hussen Abdu:** writing – original draft, writing – review and editing, formal analysis, data curation. **Endris Seid:** writing – original draft, writing – review and editing, methodology, formal analysis. **Ebrahim Msaye Assefa:** writing – original draft, methodology, writing – review and editing, software, formal analysis, data curation, supervision.

## Funding

The authors received no specific funding for this work.

## Ethics Statement

The authors have nothing to report.

## Conflicts of Interest

The authors declare no conflicts of interest.

## Transparency Statement

The lead author, Seid Mohammed Abdu, affirms that this manuscript is an honest, accurate, and transparent account of the study being reported; that no important aspects of the study have been omitted; and that any discrepancies from the study as planned (and, if relevant, registered) have been explained.

## Supporting information

S1 Appendix. Search strategies.

S2 Appendix. AQUA checklist reports.

## Data Availability

The data that support the findings of this study are available in the supplementary material of this article.
