# Supplementary material for: Prevalence of Accessory Renal Arteries in Africa: A Systematic Review and Meta‐Analysis Using Anatomical Quality Assurance (AQUA) Checklist
Source: Health Sci Rep. 2026 Jan 14;9(1):e71751. doi: 10.1002/hsr2.71751 (PMC12802860; doi:10.1002/hsr2.71751)
Supplement: Supplementary file 1 — S1 Appendix. Search strategies. [file HSR2-9-e71751-s001.docx]

**Appendix 1:** The search strategies employed in the systematic review and meta-analysis to identify relevant studies.

1. **Google Scholar:**

Search conducted on 01, April, 2024, where possible terms were searched in MeSH as well as text

Record Retrieved=160

(("prevalence"[Title/Abstract] OR "incidence"[Title/Abstract] OR "magnitude"[Title/Abstract] OR "epidemiology"[Title/Abstract] OR "proportion"[Title/Abstract] OR "burden"[Title/Abstract]) AND ("accessory renal arteries"[Title/Abstract] OR "supernumerary renal arteries"[Title/Abstract] OR "multiple renal arteries"[Title/Abstract] OR "additional renal arteries"[Title/Abstract] OR "aberrant renal arteries"[Title/Abstract])) AND (2000/1/1:2024/4/1[pdat])

1. **Search Conducted in Hinari**

Search conducted 03 April 2024

Possible terms were searched in MeSH as well as text words

Result Retrieved: 137

Summon™: ((prevalence) OR (incidence) OR (magnitude) OR (epidemiology) OR (proportion) OR (burden)) AND (("supernumerary renal arteries") OR ("accessory renal arteries") OR ("multiple renal arteries") OR ("supplementary renal arteries") OR ("additional renal arteries") OR ("aberrant renal arteries") OR (‘anatomical variation of renal arteries’))

**Search Conducted in Medline (PubMed)**

Search conducted on 01 April, 2024, where possible terms were searched in MeSH as well as text words

Record Retrieved=94

| #3 | (("prevalence"[Title/Abstract] OR "incidence"[Title/Abstract] OR "magnitude"[Title/Abstract] OR "epidemiology"[Title/Abstract] OR "proportion"[Title/Abstract] OR "burden"[Title/Abstract]) AND ("accessory renal arteries"[Title/Abstract] OR "supernumerary renal arteries"[Title/Abstract] OR "multiple renal arteries"[Title/Abstract] OR "additional renal arteries"[Title/Abstract] OR "aberrant renal arteries"[Title/Abstract])) AND (2000/1/1:2024/4/1[pdat]) | 94 |
| --- | --- | --- |
| #2 | ((((((‘accessory renal arteries’[Title/Abstract]) OR (‘supernumerary renal arteries’[Title/Abstract])) OR (‘supplementary renal arteries’[Title/Abstract])) OR (‘multiple renal arteries’[Title/Abstract])) OR (‘additional renal arteries’[Title/Abstract])) OR (‘aberrant renal arteries’[Title/Abstract])) OR (‘anatomical variation of renal arteries’[Title/Abstract]) AND (2000/1/1:2024/4/1[pdat]) | 485 |
| #1 | ((((prevalence[Title/Abstract]) OR (incidence[Title/Abstract])) OR (magnitude[Title/Abstract])) OR (epidemiology[Title/Abstract])) OR (proportion[Title/Abstract]) AND (2000/1/1:2024/4/1[pdat]) | 2087306 |

**Legend**: This table summarizes the search strategies employed to identify relevant studies for the systematic review and meta-analysis on the prevalence and patterns of accessory renal arteries (ARAs) in Africa. The search was conducted across various databases, including Google Scholar, Hinari, and Medline (PubMed), between April 1 and April 3, 2024. The strategies incorporated MeSH terms and text words focusing on ARAs, its variants, and African countries. The total number of records retrieved from each database is also provided.
