# Supplementary material for: Prevalence of Accessory Renal Arteries in Africa: A Systematic Review and Meta‐Analysis Using Anatomical Quality Assurance (AQUA) Checklist
Source: Health Sci Rep. 2026 Jan 14;9(1):e71751. doi: 10.1002/hsr2.71751 (PMC12802860; doi:10.1002/hsr2.71751)
Supplement: Supplementary file 2 — S2 Appendix. AQUA checklist reports. [file HSR2-9-e71751-s002.docx]

**S2 Appendix. Quality assessment of included studies.**

**Reviewer**: SMA & EMA, Date: May 12, 2024

**Year**: 2024.

Yes No Unclear

**Domain 1: OBJECTIVE(S) AND**

**SUBJECT CHARACTERISTICS**

1. Was (Were) the objective(s) of the study clearly defined? □ □ □
2. Was (Were) the chosen subject sample(s) and sample size □ □ □

appropriate for the objective(s) of the study? □ □ □

1. Are the baseline and demographic characteristics of the subjects

(age, sex, ethnicity, healthy or diseased, etc.) □ □ □

appropriate and clearly defined? □ □ □

1. Could the method of subject selection have in

any way introduced bias into the study?? □ □ □

**Domain 2: STUDY DESIGN**

1. Does the study design appropriately address the research question(s)? □ □ □
2. Were the materials used in the study appropriate for the

given objective(s) of the study? □ □ □

1. Were the methods used in the study appropriate for the

given objective(s) of the study? □ □ □

1. Was the study design, including methods/techniques applied in

the study, widely accepted or standard in the literature?

If “no”, are the novel features of the study design clearly described? □ □ □

1. Could the study design have in any way introduced bias into the study? □ □ □

**Domain 3: METHODOLOGY CHARACTERIZATION**

1. Are the methods/techniques applied in the study

described in enough detail for them to be reproduced? □ □ □

1. Was the specialty and the experience of the individual(s)

performing each part of the study

(such as cadaveric dissection or image assessment) clearly stated? □ □ □

1. Are all the materials and methods used in the study clearly described,

including details of manufacturers, suppliers etc.? □ □ □

1. Were appropriate measures taken to reduce

inter- and intra-observer variability? □ □ □

1. Do the images presented in the study indicate an accurate

reflection of the methods/techniques

(imaging, cadaveric, intraoperative, etc.) applied in the study? □ □ □

1. Could the characterization of methods have in any way introduced bias into the study? □ □ □

**Domain 4: DESCRIPTIVE ANATOMY**

1. Were the anatomical definition(s) (normal anatomy, variations, classifications, etc.)

clearly and accurately described? □ □ □

1. Were the outcomes and parameters assessed in the study (variation, length, diameter, etc.)

appropriate and clearly defined? □ □ □

1. Were the figures (images, illustrations, diagrams, etc.)

presented in the study clear and understandable? □ □ □

1. Were any ambiguous anatomical observations (i.e., those likely to be classified as “others”)

clearly described/depicted? □ □ □

1. Could the description of anatomy have in any way introduced bias into the □ □ □

**Domain 5: REPORTING OF RESULTS**

1. Was the statistical analysis appropriate? □ □ □
2. Are the reported results as presented in the study clear and comprehensible, □ □ □

and are the reported values consistent throughout the manuscript? □ □ □

1. Do the reported numbers or results always correspond to the number of subjects in the study?

If not, do the authors clearly explain the reason(s) for subject exclusion? □ □ □

1. Are all potential confounders reported in the study,

and subsequently measured and evaluated, if appropriate?

1. Could the reporting of results have in any way introduced bias into the study? □ □ □

**Table S1**. AQUA Checklist for included Prevalence Studies

| Authors, year | Domain 1 (n=4) | Domain 2 (n=5) | Domain 3 (n=6) | Domain 4 (n=5) | Domain 5 (n=5) | Total  (25) |
| --- | --- | --- | --- | --- | --- | --- |
| Abba S. et al 2015[1] | 2 | 5 | 4 | 5 | 3 | 19 |
| ZelalemA.etal2017[2] | 2 | 5 | 5 | 5 | 3 | 20 |
| Gebre. A. et al 2020[3] | 4 | 5 | 4 | 5 | 3 | 21 |
| Amal Y. et al 2011[4] | 4 | 5 | 5 | 5 | 3 | 22 |
| Safaa M. et al[5] | 4 | 5 | 5 | 5 | 4 | 23 |
| MugahidA.etal 2016[6] | 2 | 5 | 4 | 5 | 3 | 19 |
| MugahidA.etal 2016[6] | 4 | 5 | 4 | 5 | 3 | 21 |
| S.S.Hassanet al 2015[7] | 2 | 5 | 4 | 5 | 3 | 19 |
| Hoda M. et al 2004[8] | 4 | 5 | 5 | 5 | 3 | 22 |
| Asmaa A. et al 2019[9] | 3 | 5 | 4 | 5 | 3 | 20 |
| W. Sameh et al 2010[10] | 2 | 5 | 4 | 5 | 3 | 19 |
| Ahmed S. et al2017[11] | 3 | 5 | 4 | 5 | 3 | 20 |
| Shrifa M et al.2022[12] | 3 | 5 | 4 | 5 | 3 | 20 |
| OC Famurewa.2016[13] | 4 | 5 | 4 | 5 | 3 | 21 |
| AbayomiA.et al 2021[14] | 3 | 5 | 4 | 5 | 3 | 20 |
| Satayapa. et al[15] | 4 | 5 | 4 | 5 | 4 | 22 |
| Abderrazak B. et al[16] | 2 | 2 | 3 | 5 | 3 | 15 |

**Footnote:**

The table summarizes the quality assessment of included studies using the AQUA Checklist. Domain 1 to Domain 5 correspond to the five thematic areas outlined above: Domain 1 (Objectives and Subject Characteristics), Domain 2 (Study Design), Domain 3 (Methodology Characterization), Domain 4 (Descriptive Anatomy), and Domain 5 (Reporting of Results). The numbers in parentheses indicate the number of items assessed in each domain. Each “Yes” response scores 1 point; “No” and “Unclear” score 0. The “Total (25)” reflects the overall quality score for each study.
